# Determining the predictors of innovation implementation in healthcare: a quantitative analysis of implementation effectiveness

**DOI:** 10.1186/s12913-014-0657-3

**Published:** 2015-01-22

**Authors:** Sara R Jacobs, Bryan J Weiner, Bryce B Reeve, David A Hofmann, Michael Christian, Morris Weinberger

**Affiliations:** Public Health Research Division, RTI International, 3040 East Cornwallis Road, Post Office Box 12194, Research Triangle Park, NC 27709-2194 USA; Department of Health Policy and Management, Gillings School of Global Public Health, University of North Carolina at Chapel Hill, Chapel Hill, North Carolina USA; Cecil G. Sheps Center for Health Services Research, University of North Carolina at Chapel Hill, Chapel Hill, North Carolina USA; Lineberger Comprehensive Cancer Center, University of North Carolina at Chapel Hill, Chapel Hill, North Carolina USA; Department of Organizational Behavior, Kenan-Flagler Business School, University of North Carolina at Chapel Hill, Chapel Hill, North Carolina USA; Center for Health Services Research in Primary Care, Durham Department of Veterans Affairs, Durham, North Carolina USA

**Keywords:** Innovation, Implementation effectiveness, Implementation climate, Innovation implementation framework, Community clinical oncology program

## Abstract

**Background:**

The failure rates for implementing complex innovations in healthcare organizations are high. Estimates range from 30% to 90% depending on the scope of the organizational change involved, the definition of failure, and the criteria to judge it. The innovation implementation framework offers a promising approach to examine the organizational factors that determine effective implementation. To date, the utility of this framework in a healthcare setting has been limited to qualitative studies and/or group level analyses. Therefore, the goal of this study was to quantitatively examine this framework among individual participants in the National Cancer Institute’s Community Clinical Oncology Program using structural equation modeling.

**Methods:**

We examined the innovation implementation framework using structural equation modeling (SEM) among 481 physician participants in the National Cancer Institute’s Community Clinical Oncology Program (CCOP). The data sources included the CCOP Annual Progress Reports, surveys of CCOP physician participants and administrators, and the American Medical Association Physician Masterfile.

**Results:**

Overall the final model fit well. Our results demonstrated that not only did perceptions of implementation climate have a statistically significant direct effect on implementation effectiveness, but physicians’ perceptions of implementation climate also mediated the relationship between organizational implementation policies and practices (IPP) and enrollment (p <0.05). In addition, physician factors such as CCOP PI status, age, radiological oncologists, and non-oncologist specialists significantly influenced enrollment as well as CCOP organizational size and structure, which had indirect effects on implementation effectiveness through IPP and implementation climate.

**Conclusions:**

Overall, our results quantitatively confirmed the main relationship postulated in the innovation implementation framework between IPP, implementation climate, and implementation effectiveness among individual physicians. This finding is important, as although the model has been discussed within healthcare organizations before, the studies have been predominately qualitative in nature and/or at the organizational level. In addition, our findings have practical applications. Managers looking to increase implementation effectiveness of an innovation should focus on creating an environment that physicians perceive as encouraging implementation. In addition, managers should consider instituting specific organizational IPP aimed at increasing positive perceptions of implementation climate. For example, IPP should include specific expectations, support, and rewards for innovation use.

## Background

Healthcare organizations continuously need to implement complex innovations. This is truer now than ever, as the Patient Protection and Affordable Care Act (ACA) introduces innovative payment and delivery arrangements such as Accountable Care Organizations, bundled payments, patient-centered medical homes, and value-based purchasing [1]. Unfortunately the failure rates for implementing complex innovations are high. Estimates range from 30% to 90% depending on the scope of the organizational change involved, the definition of failure, and the criteria to judge it [2-4]. Innovations in healthcare often fail due in part to poor implementation, which can result from high uncertainty, risk, and the clinical discretion required to practice medicine. In addition, physicians tend to strongly identify with their profession compared to their organization, and may perceive a conflict between leadership goals and workforce goals [1,5]. Additional reasons for failure include misaligned incentives for adoption, un-sustained leadership, lack of support and/or training, competing priorities, and resistance to change [1,6]. Implementation failure may not only result in the loss of time and money for the organization, but can also impact the quality of care patients receive.

Theories of innovation implementation offer a promising approach to examine organizational factors that influence effective implementation [7]. Specifically, the innovation implementation framework was developed in manufacturing, although it has been increasingly applied to innovation implementation in healthcare [7-10]. To date, most of the evidence supporting its use in healthcare is qualitative in nature [8,9,11-13]. Although important, qualitative studies tend to use small sample sizes, have limited external generalizability, and present challenges in standardizing the measurement of key constructs. Currently, the majority of quantitative studies testing this framework have examined the effectiveness of technology implementation among company employees in information systems and computing organizations [14-20]. These settings are difficult to compare to healthcare because: (1) physicians experience greater professional autonomy, and (2) the process of implementing computing technology offers greater standardization than delivering clinical care or implementing innovative care delivery models.

It is important to quantitatively examine the innovation implementation framework as it will allow for a more precise examination of proposed hypotheses as well as will allow researchers to compare results across settings, samples, and innovations. A quantitative analysis also allows researchers to control for other explanatory variables that may predict implementation effectiveness, which is difficult to do with qualitative research. Therefore, we quantitatively tested the innovation implementation framework in the National Cancer Institute (NCI)’s Community Clinical Oncology Program (CCOP), a provider-based research network focused on the enrollment of patients in cancer clinical trials [21]. Using both survey and archival data, we examined the hypothesized relationship among core constructs of the model using structural equation modeling (SEM). Specifically we sought to investigate the role of implementation climate and organizational implementation policies and practices (IPP) in determining the effectiveness of innovation implementation.

Our research extends the literature surrounding the innovation implementation framework, as it is the first study to quantitatively test the innovation implementation framework in a healthcare context. In addition, the framework tested in this paper focuses on implementation among individual physicians, rather than at the organizational or group level which is common when testing innovation implementation models [14-16,18,22]. We were most interested in examining *individual* physician participation in CCOP because of the significant variation that occurs in enrolling patients in cancer clinical trials. Although all physicians agree to participate in CCOP at some level, the number of patients enrolled by CCOP physicians in 2011 ranged from 0 to 88 patients per physician, with over 40% of physicians enrolling zero patients. Examining the innovation implementation framework among individuals is a critical advancement given many innovations in healthcare are implemented voluntarily by individual physicians. In addition, it is important to adapt an organizational level theory to examine individual physician enrollment because although physicians have a level of autonomy not typically experienced in other non-healthcare settings, they are nonetheless embedded in a clinical context. Organizational contextual factors beyond an individual physician’s willingness to participate or their desire to implement an innovation may impede their ability to do so. Therefore, this study also has important practical implications for implementing innovations in complex, rapidly changing healthcare organizations.

## Methods

### Conceptual framework

**Figure 1:**
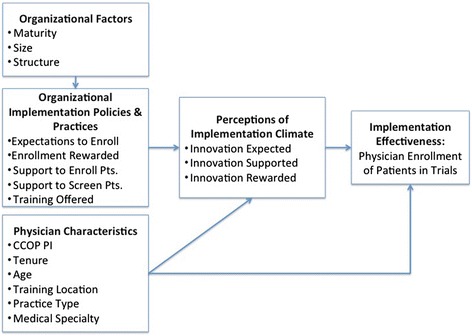
**The impact of implementation climate on physician enrollment.**

The conceptual model for this study is based on Klein and colleagues’ innovation implementation framework which specifics the antecedents of complex innovation implementation [7,16]. The framework postulates that implementation effectiveness, or the consistency and quality of innovation use, results from both organizational implementation policies and practices (IPP) and individual climate perceptions (Figure 1). IPP are the formal strategies organizations utilize to put the innovation into use, while implementation climate is the extent to which organizational members perceive that an innovation is expected, supported, and rewarded by their organization [7,9,16]. Specifically, the authors suggest that IPP are the antecedents of climate, while individuals’ interpretive perceptions of climate ascribe meaning to the policies and practices [23]. Therefore, how physicians view their organization in terms of encouraging innovation implementation is determined by IPP. In addition, these perceptions predict the number of patients each physician will enroll in a cancer clinical trial (i.e., implementation effectiveness). Therefore, our first hypothesis is as follows:

1. IPP will have a positive indirect effect on implementation effectiveness operating through implementation climate perceptions.

A strong implementation climate ensures organizational members, or in this case physicians, have the skills and support needed to implement the innovation, incentives to participate are in place, and implementation obstacles are limited. In this setting, strong perceptions of implementation climate should directly lead to stronger implementation effectiveness (higher patient enrollment among physicians). We expect higher patient enrollment among physicians with strong implementation climate perceptions because perceptions that a CCOP institutes enrollment expectations, provides support for the enrollment of patients, as well as recognizes enrollment efforts should encourage physicians to enroll more patients in cancer clinical trials. Therefore, our second hypothesis is as follows:

H2: Perceptions of implementation climate will have a direct positive effect on implementation effectiveness.

Given we are interested in examining an innovation implemented by individual physicians, we needed to modify the innovation implementation framework to ensure that the constructs were relevant at the individual level [24]. For example, we were interested in examining *individual* physician participation in CCOP because of the significant variation that occurs in enrolling patients in cancer clinical trials. Thus, we added control variables such as physician characteristics (i.e., physician age, years of experience, specialty, practice type, training location, and CCOP Principal Investigator (PI) status), as we believed these characteristics would both influence physicians’ perceptions of climate and their ability to enroll patients in cancer clinical trials. For example, experience and age may influence perceptions, as older and more experienced physicians may perceive they have access to more resources. Physician characteristics may also impact enrollment, as more experienced physicians or CCOP PIs may be more familiar with clinical trials and enroll more patients. Therefore, our third hypothesis is as follows:

H3: Physician characteristics will have both direct and indirect effects on implementation effectiveness operating through perceptions of implementation climate.

Lastly, we included organizational control factors, such as structure, years in existence, and size in our model. For example, we believed larger organizations that are part of a cancer center or research institute may have more resources to encourage innovation implementation compared to smaller, non-profit independent organizations. In addition, we believed that CCOPs that had been in existence longer would likely have more resources and thus provide more trainings and offer greater incentives for physicians to enroll patients. Therefore, our fourth hypothesis is as follows:

H4: Organizational factors will have an indirect effect on implementation effectiveness operating through IPP and perceptions of implementation climate.

### Study setting

The study was conducted in NCI’s CCOP network. In brief, the goals of the CCOP network are to advance the evidence-base by conducting research in clinical settings where most people receive their care, and translate results into better care [21,25,26]. The CCOP network is a joint venture between NCI’s Division of Cancer Prevention, who provides overall direction and funding for community hospitals and practices to participate in clinical trials, selected cancer centers and clinical cooperative groups (CCOP research bases), who design the trials, and community-based physicians and hospitals (CCOP organizations) care, who assist with patient enrollment, data collection, and dissemination of study findings [21,25,26]. CCOP organizations are generally composed of a physician CCOP PI who provides local program leadership, a team of support staff, as well as affiliated physicians who enroll patients in NCI-sponsored cancer clinical trials [8,12]. CCOP-affiliated physicians include specialized oncologists (e.g., hematological, surgical and radiation oncologists), general medical oncologists, and other medical specialists (e.g., urologists, gynecologists, and gastroenterologists).

In 2011, when the study was conducted, the CCOP network consisted of 47 CCOP organizations across 28 states, the District of Columbia, and Puerto Rico and included 400 hospitals and 3,520 community physicians. CCOP organizations consisted on average of 10 community hospitals or physician practices and 48 physicians. This study was determined to be exempt from review by the Institutional Review Board at the University of North Carolina at Chapel Hill.

### Data sources and data collection procedures

The data for this cross-sectional study were obtained from four sources. First, the 2011 CCOP Annual Progress Reports, submitted in March 2012, provided data on physicians’ enrollment activities during the period from June 1, 2011 to February 29, 2012. The reports were used to determine the outcome, physician enrollment of patients in trials. Each March, every CCOP submits a progress report to NCI detailing the previous nine-month’s research and enrollment activities. The report includes standardized questions regarding the allocation of CCOP resources, staffing assignments, total cancer patient volume, the number of open cancer clinical trials, the total number of patients each CCOP enrolls, as well as the total number of patients each individual CCOP-affiliated physician enrolls. These reports are not publically available. We received permission from the NCI Division of Cancer Prevention to access and use these data for our study.

This study also used the 2011 CCOP Physician Survey and the 2011 CCOP Administrator Survey, which were both designed and administered as part of a larger NCI-funded-study (5R01CA124402). Both surveys were reviewed by the Institutional Review Board at the University of North Carolina at Chapel Hill and included instructions for completing the survey including that completion of the respective survey connoted consent to be a participant in the study. The goal of the physician survey was to learn more about physician participation in the CCOP program, while the administrator survey collected information regarding CCOP policies and procedures. The physician survey included specific questions regarding physician perceptions surrounding expectations for enrollment, research support provided by the CCOP, ability to provide input on which clinical trials to open, how well he or she is kept informed of CCOP activities, recognition received from the CCOP, as well as attitudes regarding the importance of cancer clinical trials. The survey specifically supplied data on CCOP physicians’ perceptions of implementation climate.

The sampling frame for the physician survey included all CCOP-affiliated physicians eligible to enroll patients to clinical trials. Between October 2011 and January 2012, we surveyed 817 physicians using a random sample stratified across all 47 CCOPs. On average, 17 physicians per CCOP were surveyed. One week after sending potential respondents a postcard announcing the survey and highlighting its importance to NCI, physicians were sent a cover letter explaining the goals of the survey, the survey itself, a self-addressed and stamped return envelope, and a $50 Visa gift card as an incentive to complete the survey. Physicians were also able to complete the survey online via a unique access code provided in the mailing. A thank-you or reminder postcard was then sent the following week. Approximately three weeks after the first mailing, non-respondents received a second copy of the survey, cover letter, and return envelope. Lastly, we contacted CCOP PIs and administrators to email the non-responding physicians affiliated with their CCOP requesting them to complete the survey. The final sample included 485 physicians (59.4% of physicians surveyed). On average, we received responses from 10 physicians per CCOP organization.

The administrator survey included questions relating to the CCOP’s organizational structure and size, performance management, education and trainings, protocol selection practices, research support, and staffing. The survey specifically supplied data on CCOP’s IPP and the organizational control factors. The survey was completed by 100% of CCOP administrators. The vast majority of administrators completed the survey at the 2011 annual CCOP meeting, held each September at NCI. We followed up via email with administrators that did not complete the survey in person. Any remaining surveys were completed between October 2011 and January 2012.

Lastly, the 2012 American Medical Association (AMA) Physician Masterfile provided data for the physician characteristics. Established by the AMA in 1906, the Physician Masterfile includes current and historical data for more than 1.4 million physicians, residents, and medical students in the U.S, including data on demographics, specialty, experience, medical school training, and residency. These data are available for purchase from the AMA.

### Measures

The outcome of this study was implementation effectiveness, which was operationally defined as the number of patients that each physician enrolled in cancer clinical trials in 2011. The NCI uses this objective, outcome-focused measure as the primary means of determining CCOP physician performance, as do other studies examining CCOP performance [27].

#### Key constructs

The organizational implementation policies and practices (IPP) construct included five measures from the CCOP Administrator Survey, as this survey was focused on CCOP level policies and practices that would impact all physicians affiliated with that CCOP. As discussed, IPP are the formal strategies organizations utilize to put the innovation into use; they include objective assessments of policies and practices the organization has in place. To measure formal expectations for enrollment, administrators were asked whether their CCOP expects physicians to enroll a minimum number of patients in clinical trials. Three measures addressed formal CCOP support for enrollment activities: 1) proportion of physicians for whom CCOP staff members routinely screen patient charts for potentially eligible patients; 2) proportion of physicians for whom CCOP staff members routinely assist with enrollment; and 3) whether or not CCOP sponsor any events where physicians could learn about the latest developments in cancer research, treatment, prevention, or control. Lastly, to assess formal rewards, administrators were asked whether the CCOP provides some form of recognition to physicians with high levels of enrollment to NCI-sponsored trials.

The perceptions of implementation climate construct included six measures from the CCOP Physician Survey that were consistent with prior studies examining perceptions of implementation climate [7,8,12]. As discussed, implementation climate is the extent to which organizational members *perceive* that an innovation is expected, supported, and rewarded by their organization. Two measures addressed whether physicians perceived they were: 1) expected to enroll a certain number of patients in trials and 2) expected to help the CCOP meet its patient enrollment goals. Two measures addressed whether physicians perceived they get the support they needed to: 1) identify potentially eligible patients and 2) enroll patients in trials. Lastly, two measures addressed whether physicians perceived they get: 1) recognition and 2) appreciation when they enroll patients in trials. For all measures, physicians could respond *disagree*, *somewhat disagree*, *neither agree nor disagree*, *somewhat agree*, or *agree*.

#### Control variables

We also included both physician characteristics and CCOP organizational factors as controls. Physician characteristics included age, whether or not they trained in the United States, self-designated medical specialty (e.g., hematologist oncologist, surgeon, radiological oncologist, general oncologist, non-oncologist specialist), and whether or not the physician was the CCOP PI. We also included the practice arrangement (i.e., hospital-, group-, or solo- based) and how long the physician had been in clinical practice. The organizational factors included CCOP organizational structure (e.g., hospital cancer center or cancer service line, research institute, department or center, separate non-profit organization), size (i.e., number of locations patients can enroll in a clinical trial), and how long the CCOP has been in existence.

### Data analysis

#### Structural equations modeling

**Figure 2:**
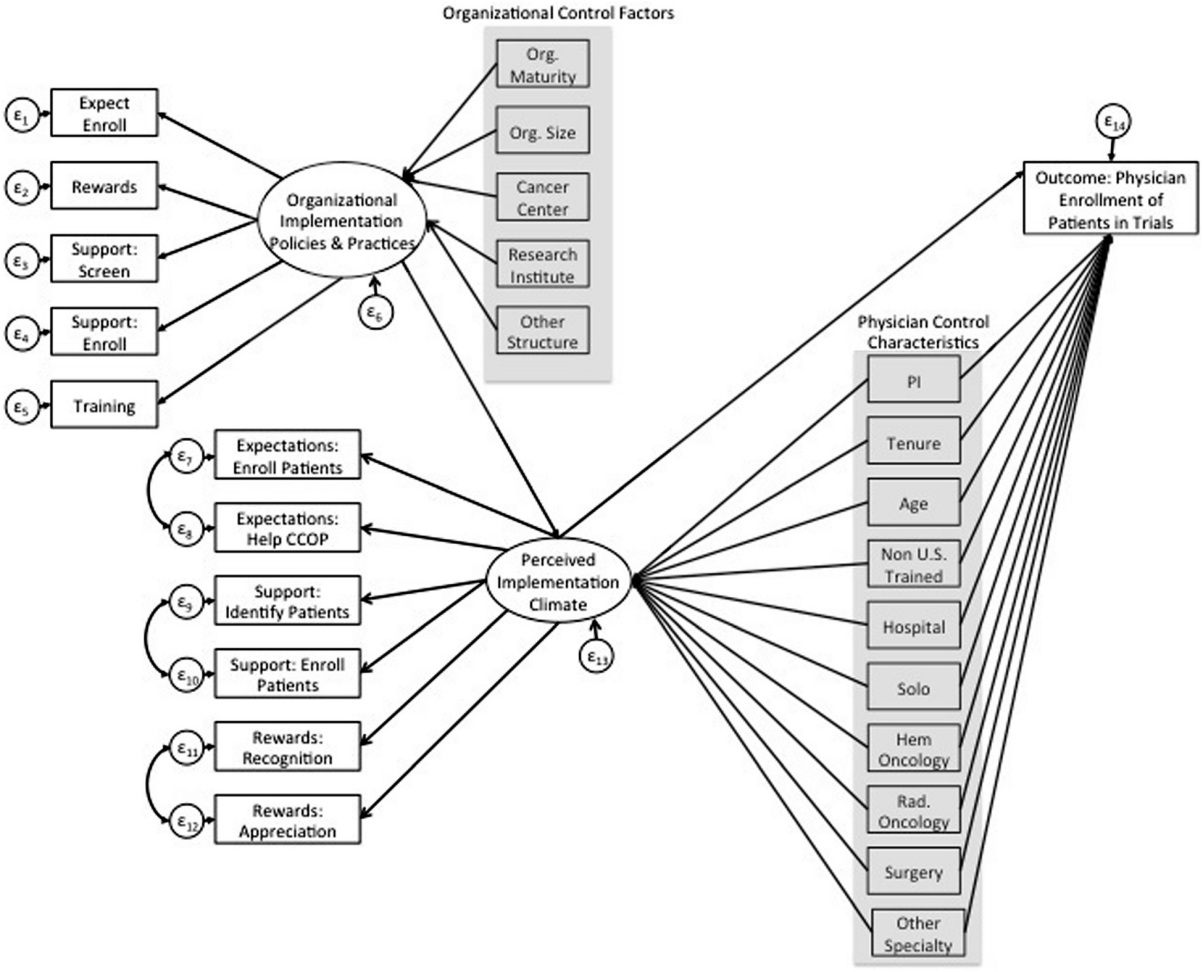
**Original proposed SEM model.** Note: Highlighted variables represent organizational control factors and physician control characteristics.

SEM with maximum likelihood estimation was used to simultaneously test whether perceptions of implementation climate mediates the relationship between IPP and implementation effectiveness. SEM is composed of multivariate regression models and can be used to estimate proposed causal relationships [28-30]. We used confirmatory SEM to test the hypothesized pathways among implementation factors represented in Figure 2 by comparing how well this proposed structure fits the observed data. We selected SEM because it allowed us to test for constructs that are not directly assessed, but are instead composed of observed indicators representing the constructs of interest (e.g., IPP, perceptions of implementation climate).

The goal of SEM is to achieve a well-fitting model based on theory [28-30]. Therefore, a priori, we believed that the two measures that composed each of the three components of perceptions of implementation climate (i.e., support, reward, expectations) would co-vary higher with each other than with the other measures representing the other components. For example, the two measures that compose expectations, physicians are expected to enroll a certain number of patients and are expected to help the CCOP meet its goals, likely share common variation that is not explained by any of the proposed relationships in the model likewise with the two measures that compose support and the two that compose rewards. In total we added three co-variances. We also elected to use clustered robust standard errors to account for clustering of physicians within the 47 CCOPs.

We then evaluated model fit using the Comparative Fit Index (CFI) and the Tucker-Lewis Index (TLI). CFI and TLI values range from 0 to 1, with values ≥ 0.90 representing adequate fit [28,30]. We also examined the root mean square error of approximation (RMSEA) and the associated confidence interval and p-value. RMSEA values < 0.06 and an upper bound of the confidence interval < 0.1 are considered acceptable. Next, we examined the standardized root mean squared residuals (SRMR), with values < 0.08 considered acceptable fit [30].

Based on these fit statistics for the original model, we elected to re-specify our original model to improve its fit. SEM is an iterative process in which model fit is improved by using theory and modifications indices either to add additional pathways between variables or to allow items to co-vary [28-30]. Modification indices are the minimum that the chi-square statistic is expected to decrease if the corresponding parameter is no longer assumed to be fixed at zero [28]. We added four additional co-variances to the original model. With the addition of each error-term co-variance, we tested whether model fit improved by examining the baseline model against the new model using the Lagrange multiplier test and fit statistics.

Once we achieved a well fitting SEM model, we evaluated our model by testing the significance of all standardized estimates. To examine standardized direct and indirect effects, we used bootstrapping with 95% confidence intervals on 1,000 bootstrap estimates. We elected to use bootstrapping to correct for non-normality, given the power of the joint test of two pathways in mediation analysis is larger than the power of the test of their product when using the usual z test and the associated confidence intervals for the product [31]. This appears to be due to the non-normality of the product. Specifically, indirect effects are the product of the two regression coefficients. For example, if X predicts Y and Y predicts Z, then the indirect effect of X on Z equals the product of the two regression coefficients (X on Y and Y on Z). Lastly, to ensure the validity of our SEM results, we checked our results using negative binomial regression analysis with clustered robust standard errors. Analyses were performed using Mplus 7.

## Results

### Descriptive statistics

The final sample for this study included 481 physicians with complete data on the 2011 CCOP-Affiliated Physician Survey (Table 1). The vast majority of the sample was male (74%), White non-Hispanic (75%), practiced in a group practice (78%), and trained in the U.S (80%). The mean age was approximately 53 years old and physicians on average had been in practice approximately 26 years. Over 70% were oncology-based specialists and 9% were the CCOP PI. Physicians on average enrolled close to five patients in a cancer clinical trial in 2011. In addition, physicians generally agreed that their CCOP encouraged implementation. The average response on all six questions relating to implementation climate was 3.6 on a scale of one to five. Physicians on average rated the two reward items the lowest (Item 1: 3.2; Item 2: 3.4) and the expectation and support items more favorably (Expectations item 1 & 2: 3.4 and 4.2; support items 1 & 2: 3.8 and 4.1). The only significant (p < 0.05) differences between survey responders and non-responders were that responders enrolled more patients per year (4.7 versus 3.4), were more likely to be a surgeon (10% versus 5%), and were less likely to be a non-specialized general oncologist (11% versus 24%).

**Table 1:** **Descriptive statistics CCOP physicians**

**CCOP physician survey respondents n = 481**			
	**Mean or proportion of sample**	**Standard deviation**	**Range**
***Outcome***			
2011 Patient Enrollment	4.7*	8.1	0, 62
***Descriptive variables***			
**Gender**			
*Male*	74%		
*Female*	26%		
**Race**			
*White*	75%		
*Asian*	15%		
*African-American*	1%		
*Other*	9%		
***Perceptions of implementation climate***			
**Expectations:** Enroll Patients	3.4	1.5	1,5
**Expectations:** Help CCOP	4.2	1.1	1,5
**Support:** Identify Patients	3.8	1.3	1,5
**Support:** Enroll Patients	4.1	1.2	1,5
**Rewards:** Recognition	3.2	1.3	1,5
**Rewards:** Appreciation	3.3	1.3	1,5
***Physician characteristics included in model***			
**Age**	52.6	9.8	34,82
**Practice type**			
*Group Practice*	78%		
*Hospital-Based*	12%		
*Solo Practice*	4%		
*Other/None Listed*	6%		
**Training location**			
*U.S Trained*	80%		
*Non U.S Trained*	20%		
**Tenure (Yrs. in practice)**	25.7	10.1	8, 57
**Medical specialty**			
*Hematology Oncology*	40%		
*Radiation Oncology*	21%		
*Other Specialty*	18%		
*Medical Oncology*	11%*		
*Surgery*	10%*		
**Principal investigator**	9%		

*Indicates significant difference between survey respondents and non-survey respondents.

*Other race* includes American Indian, Native Hawaiian/Pacific Islander, More than one race, or unknown.

*Hematology oncology* includes blood banking, hematology oncology, hematology.

*Radiation Oncology* includes diagnostic radiology, nuclear medicine, radiation oncology, radiology, vascular and interventional radiology.

*Other specialist* includes general practice, gynecological oncology, pediatrics, pediatric hematology, cardiovascular disease etc.

*Surgery* includes colon and rectal surgery, critical care sugary, general surgery, neurological surgery, surgical oncology, urological surgery etc.

For the CCOPs (n = 47), the average number of years in existence was over 25 (Table 2). The average size, as determined by the number of locations a patient could enroll in a clinical trial, was 14 and the majority of CCOPs were a hospital cancer center or cancer service line (40%), although 30% were a separate non-profit organization and 24% were a research institute, department, or center. The majority of CCOPs did not institute a minimum number of patients physicians should enroll per year (65%), did not offer trainings or events where physicians could learn about the latest developments in cancer research (58%), but did provide some form of recognition to physicians with high levels of enrollment (62%). In addition, about 50% of physicians within a CCOP had support staff members help screen patient charts for potentially eligible patients and assist with enrollment.

**Table 2:** **Descriptive statistics CCOP organizations**

**CCOP Administrator survey respondents n = 47**			
	**Mean or proportion of sample**	**Standard deviation**	**Range**
***Organizational implementation context***			
**Expectations:** Enroll Patients			
*Yes*	35%		
*No*	65%		
**Support:** Identify Patients	0.51	0.32	0,1.5
**Support:** Enroll Patients	0.50	0.32	0,1.5
**Support:** Training			
*Yes*	32%		
*No*	68%		
**Rewards:** Recognition			
*Yes*	62%		
*No*	38%		
***Organizational factors included in model***			
**Organizational maturity**	25.5	6.2	8,30
**Organizational size**	14.3	15.6	2,87
**Organizational structure**			
*Cancer Center*	41%		
*Research Institution*	24%		
*Separate Organization*	30%		
*Other*	5%		

#### SEM measurement analysis

**Figure 3:**
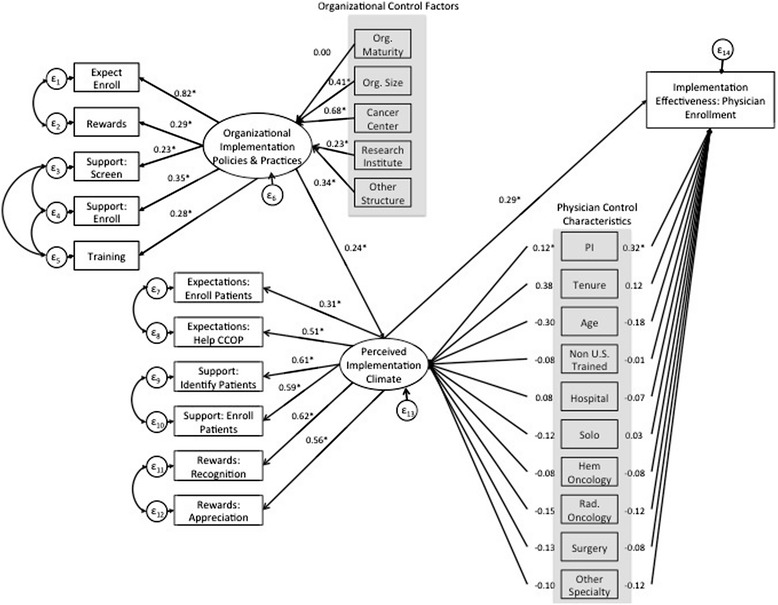
**Final SEM model with standardized estimates.** Note: Highlighted variables represent organizational control factors and physician control characteristics.

The fit statistics and modification indices for the fixed parameters of the original model suggested that we re-specify the model to improve fit (CFI = 0.846 TLI = 0.815; RMSEA = 0.068; SRMR = 0.052) (Figure 2). Therefore, we added four post-hoc modifications that were theoretically justified and improved model fit (Figure 3). For these modifications, we allowed the error terms of the following measures to co-vary higher than with other variables. For example, the percentage of doctors supported in screening and enrolling patients, likely share common variation that is not explained by any of the proposed relationships in the model.

1. The percentage of doctors supported in screening and enrolling patients: The same support staff generally preform both functions within a CCOP.
2. Trainings offered with the percentage doctors who are supported in screening patients: The number of trainings offered relates to the number of support staff available.
3. Trainings offered with the percentage doctors who are supported in enrolling patients: The number of trainings offered relates to the number of support staff available.
4. Rewards with expectations for enrollment: CCOPs that provide incentives may also be more likely to have expectations for enrollment.

With the addition of each error-term co-variance, we tested whether model fit improved by examining the baseline model against the new model using the Lagrange multiplier test and fit statistics. Figure 3 provides a graphic version of the final standardized bootstrapped SEM results. Standardized bootstrapped total, direct, and indirect effects are provided in Table 3. Overall, we achieved a final well-fitting model (CFI = 0.933; TLI = 0.918; RMSEA = 0.045; SRMR = 0.048) and explained approximately 24% of the total variation in implementation effectiveness.

**Table 3:** **Standardized total, direct, and indirect effects**

	**Total effect**	**Direct effect**	**Indirect effect**
***Outcome: Enrollment in NCI-Sponsored Cancer Clinical Trials in 2011***			
**Perceptions of Implementation Climate**	0.285*	0.285*	N/A
**Organizational Implementation Policies and Practices (IPP)**	0.069*	N/A	0.069*
*Age*	−0.264*	−0.179	−0.085
*Hospital-Based^*	−0.043	−0.066	0.023
*Solo Practice^*	−0.001	0.034	−0.035
*Non U.S. Trained*	−0.035	−0.011	−0.024
*PI*	0.356*	0.322*	0.034
*Tenure*	0.224	0.117	0.107
*Oncology* ^*+*^	−0.097	−0.075	−0.022
*Radiation Oncology* ^*+*^	−0.162*	−0.120	−0.042
*Surgery* ^*+*^	−0.114	−0.077	−0.037
*Other Specialty* ^*+*^	−0.147*	−0.120	−0.027
*Organizational Size*	0.028*	N/A	0.028*
*Structure: Hospital Cancer Center* ^*++*^	0.047*	N/A	0.047*
*Structure: Research Institute* ^*++*^	0.016*	N/A	0.016*
*Structure: Other* ^*++*^	0.024*	N/A	0.024*
*Organizational Maturity*	0.000	N/A	0.000

Model Fit Statistics: CFI = 0.933; TLI = 0.918; RMSEA = 0.045; SRMR = 0.048.

Note: Total effects is the sum of direct and indirect effects.

Note: Indirect effects are the product of the regression coefficients leading to the outcome. For example for OIPP, OIPP predicts perceptions and perceptions predicts enrollment. The indirect effect and subsequently the total effect of OIPP on enrollment equals the product of the two regression coefficients (From Figure 3) 0.243*0.285 = 0.069.

*Statistically Significant (p < 0.05).

^Compared to Group Practice.

^+^Compared to General Non-Specialized Oncology.

^++^Compared to Separate Non-Profit Structure.

Regarding the IPP construct, the majority of the construct was determined by expectations for enrollment (β = 0.82), although all five measures were statistically significant (p < 0.05). In addition, regarding the perceived implementation climate construct, all six measures were statistically significant determinants of the construct (p < 0.05). The largest determinants were perceptions regarding the organization’s recognition and appreciation of enrollment activities (β = 0.62; β = 0.56) as well as perceptions regarding support provided to screen and enroll eligible patients (β = 0.61; β = 0.59) (Figure 3).

#### SEM structural analysis: results from hypotheses testing

**Figure 4:**
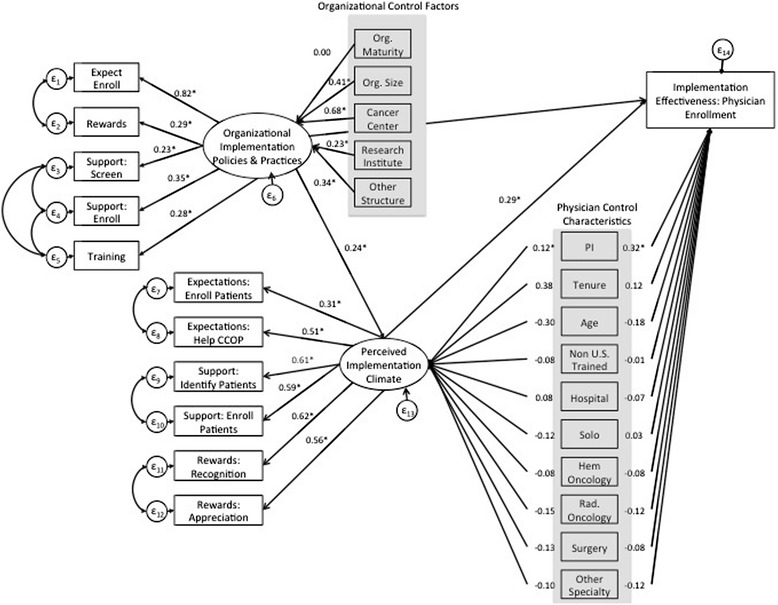
**Alternative SEM model with standardized estimates.** Note: Highlighted variables represent organizational control factors and physician control characteristics.

Hypothesis one was supported, as physicians’ perceptions of implementation climate mediated the relationship between IPP and enrollment. This means there was a significant indirect effect between IPP and enrollment operating through perceptions of implementation climate (indirect effect = 0.069; p = 0.01) (Table 3). Although our final model (Figure 3) indicated that implementation climate fully mediated the relationship between IPP and implementation effectiveness, this model did not test for partial mediation where IPP would also directly influence implementation effectiveness in addition to impacting perceptions of implementation climate. In our study setting, we could also envision scenarios where IPP also directly influenced implementation effectiveness. For example, in addition to helping to shape perceptions of implementation climate, the number of staff available at each CCOP to screen and enroll patients might also directly determine the number of patients a physician was able to enroll in a clinical trial. Although it is not included in the innovation implementation framework, we decided to also test an alternative model, where IPP also had a direct pathway to implementation effectiveness (Figure 4). This alternative model fit the data almost identically to our final model (CFI = 0.934; TLI = 0.918; RMSEA = 0.045; SRMR = 0.047). The standardized estimates are also the same as our final model. The results of the alternative model do indicate, however, that there also was a significant direct effect of IPP on implementation effectiveness (direct effect = 0.10; p = 0.04). Therefore, perceptions of implementation climate likely only partially mediated the relationship between IPP and implementation effectiveness, as there was also a direct relationship between the two constructs as observed in the alternative model.

Returning to our final model, hypothesis two was also supported as perceptions of implementation climate had a statistically significant direct effect on implementation effectiveness (direct effect = .285; p < 0.00). Hypothesis three was partially supported given CCOP PI status, age, radiological oncologists, and non-oncologist specialists significantly influenced enrollment while training location, tenure, practice location, and physicians who are surgeons, and hematologists (compared to non-specialized oncologists) did not directly influence implementation effectiveness. There was no evidence, however, that any of the physician characteristics significantly influenced implementation effectiveness through their effect on perceptions of implementation climate. Lastly, hypothesis four was also partially supported as organizational size and structure indirectly influenced implementation effectiveness through IPP and implementation climate. However, organizational maturity did not significantly influence implementation effectiveness through its effect on IPP and perceptions of implementation climate. The robustness check of our SEM results using negative binomial regression analysis with clustered robust standard errors confirmed our main findings.

## Discussion

### Theoretical significance

Overall, our results quantitatively confirmed the main relationship outlined in the innovation implementation framework between IPP, perceptions of implementation climate, and implementation effectiveness among individual physicians [7]. It is often difficult to test this framework quantitatively given the large sample of participants and organizations required. Although the framework has been discussed within healthcare organizations before, the studies have been predominately qualitative in nature. For example, Helfrich and colleagues demonstrated the relationship between IPP, implementation climate, and effectiveness using comparative case studies of four cancer clinical research networks implementing new programs in cancer prevention and control research [9]. Similar results have also been confirmed in other settings. Sawang and Unsworth confirmed a similar framework where implementation climate mediated the role of IPP and implementation effectiveness among small and medium businesses implementing different innovations in Australia [18].

In addition, our results demonstrate the potential for using the innovation implementation framework in healthcare to explain *individual* level innovations. Our results confirmed that individual perceptions of implementation climate should also be composed of measures relating to expectations, support, and rewards, as suggested in the innovation implementation framework [7]. Although, the framework suggests that implementation climate should be assessed at the group level as an aggregation of shared perceptions, our results demonstrate that implementation climate can also be measured at the individual level using the same theoretical construct. Demonstrating the utility of the framework among individuals is important given many innovations and evidence-based practices in healthcare are implemented by individual physicians on a voluntary basis. For example, the use of novel therapies often only require implementation by a single physician, not collective, coordinated implementation among multiple individuals in an organization.

Our findings are also relevant to other implementation theories and frameworks. For example, our results can be used in conjunction with the Consolidated Framework for Implementation Research (CFIR). The CFIR offers guidance as to the possible predictors of implementation effectiveness, such as intervention characteristics, factors at the system and organizational levels, and characteristics of the individuals implementing the innovation [32]. The CFIR does not, however, provide rationale as to *why* some domains may be more relevant than others and *how* they are related in certain circumstances. Therefore our results could be useful in selecting relevant constructs from the CFIR to examine implementation effectiveness of individually driven innovations in a healthcare setting. For example, as part of the inner setting or organizational level construct, the CFIR includes organizational characteristics, such as size and structure, which we found to be important determinants of organizational IPP. The inner setting also includes policies and practices related to implementation climate, such as organizational incentives and rewards, clearly communicated goals and feedback, available resources, and access to information through trainings, all of which were included in our model as IPP and/or in physicians’ perceptions of implementation climate. Lastly, the CFIR also includes characteristics of the individual such as tenure, age, and experience. Our research indicates how these constructs not only relate to one another, but how they also impact implementation effectiveness.

### Practical implications

Our results demonstrated that implementation climate perceptions partially mediated the relationship between IPP and implementation effectiveness. Therefore, the policies and practices an organization has in place to encourage innovation implementation may be most effective if the intended users *perceive* these policies and practices as supportive. Although there was a significant direct effect between IPP and implementation effectiveness, over a third of the total effect of IPP on implementation effectiveness resulted from the *indirect* effect of IPP on implementation effectiveness operating through perceptions of implementation climate. Therefore, even with supportive IPP in place, implementation could still fail if the intended users do not feel the effects of these IPP as encouraging implementation. In addition, the direct relationship between *implementation climate* and implementation effectiveness was almost three times greater than the relationship between *IPP* and implementation effectiveness. Thus, managers looking to increase implementation effectiveness of an innovation should focus on creating an environment that physicians perceive as encouraging implementation. For example, ensuring physicians *feel* that they are supported and perceive that they get what they need to effectively implement an innovation is more important than having a certain number of staff available or offering trainings in terms of encouraging implementation.

We also proposed that personal characteristics would have both direct effects on implementation effectiveness as well as indirect effects on implementation effectiveness operating through climate perceptions. However, we only found direct effects for some of the personal characteristics. Although we did find that status as the CCOP PI had a significant effect on climate perceptions, the indirect effect operating through climate perceptions on implementation effectiveness was not significant. Therefore intended users that are leaders or innovation champions may have more positive perceptions of climate compared to non-leaders. Overall, however, these results indicate that climate perceptions were mostly determined by IPP. Our findings suggest that there may be alternative ways in which personal characteristics relate to implementation effectiveness. For example, personal characteristics may have an influence on fit between the innovation and the organizational members’ values [7]. Perhaps, more experienced physicians or physicians that have been at the organization longer perceive the innovation as more congruent with their individual values and therefore use the innovation in a more consistent and high-quality way. This should be tested in future studies. In addition, it is possible that personal characteristics *moderate* the relationship between implementation climate and implementation effectiveness. For example, experience, or status as an innovation leader may strengthen the effect of perceptions of implementation climate on implementation effectiveness. So, experience or status as a leader would intensify the relationship between implementation climate and implementation effectiveness. Therefore, future studies should examine other potential relationships between personal characteristics and implementation effectiveness.

### Limitations

There are several limitations of our study. First, we only included physicians who participated in CCOP in our study. In addition, CCOP Physician Survey respondents significantly enrolled more patients per year than survey non-respondents. Thus, we need to be careful in generalizing our results to all CCOP physicians as well to other types of physicians. Our findings might be most relevant to encourage *active* participation in settings where innovation use is voluntary. The framework should also be tested in settings implementing a variety of innovations especially where participation or implementation is mandatory. Second, our study is cross-sectional and only represents a single point in time. Future studies should consider examining implementation climate over the course of implementation to better understand how climate may vary over time or among different groups within a single organization. Lastly, although many innovations in healthcare are focused on the individual, we did not test the framework at the intended unit of analysis, the organizational level. Therefore future work should also consider investigating this framework at the organizational or practice level by aggregating implementation climate perceptions if possible.

## Conclusion

Through this analysis we were able to extend the literature concerning the use of the innovation implementation framework as well as provide practical suggestions for managers considering implementing an innovation. Our study not only offers quantitative evidence that perceptions of implementation climate mediates the relationship between IPP and implementation effectiveness, but it also demonstrates the utility of adapting an implementation framework to explain innovations implemented among individuals. The majority of our hypotheses were supported, thus demonstrating the importance of physicians’ perceptions of implementation climate in determining implementation effectiveness. Therefore, managers looking to increase innovation implementation effectiveness should consider fostering a strong implementation climate through supportive IPP to encourage innovation use.
